# Proteome and transcriptome profiling of equine myofibrillar myopathy identifies diminished peroxiredoxin 6 and altered cysteine metabolic pathways

**DOI:** 10.1152/physiolgenomics.00044.2018

**Published:** 2018-10-05

**Authors:** Stephanie J. Valberg, Sudeep Perumbakkam, Erica C. McKenzie, Carrie J. Finno

**Affiliations:** ^1^McPhail Equine Performance Center, Department of Large Animal Clinical Sciences, Michigan State University, East Lansing, Michigan; ^2^Department of Population Sciences, University of Minnesota, St. Paul, Minnesota; ^3^Department of Large Animal Clinical Sciences, Michigan State University, East Lansing, Michigan; ^4^Department of Clinical Sciences, Carlson College of Veterinary Medicine, Oregon State University, Corvallis, Oregon; ^5^Department of Population Health and Reproduction, University of California Davis, Davis, California

**Keywords:** antioxidant, horse, myopathy, proteome, RNA-Seq, transcriptome

## Abstract

Equine myofibrillar myopathy (MFM) causes exertional muscle pain and is characterized by myofibrillar disarray and ectopic desmin aggregates of unknown origin. To investigate the pathophysiology of MFM, we compared resting and 3 h postexercise transcriptomes of gluteal muscle and the resting skeletal muscle proteome of MFM and control Arabian horses with RNA sequencing and isobaric tags for relative and absolute quantitation analyses. Three hours after exercise, 191 genes were identified as differentially expressed (DE) in MFM vs. control muscle with >1 log_2_ fold change (FC) in genes involved in sulfur compound/cysteine metabolism such as cystathionine-beta-synthase (*CBS*, ↓4.51), a cysteine and neutral amino acid membrane transporter (*SLC7A10*, ↓1.80 MFM), and a cationic transporter (SLC24A1, ↓1.11 MFM). In MFM vs. control at rest, 284 genes were DE with >1 log_2_ FC in pathways for structure morphogenesis, fiber organization, tissue development, and cell differentiation including > 1 log_2_ FC in cardiac alpha actin (*ACTC1* ↑2.5 MFM), cytoskeletal desmoplakin (*DSP* ↑2.4 MFM), and basement membrane usherin (*USH2A* ↓2.9 MFM). Proteome analysis revealed significantly lower antioxidant peroxiredoxin 6 content (PRDX6, ↓4.14 log_2_ FC MFM), higher fatty acid transport enzyme carnitine palmitoyl transferase (CPT1B, ↑3.49 MFM), and lower sarcomere protein tropomyosin (TPM2, ↓3.24 MFM) in MFM vs. control muscle at rest. We propose that in MFM horses, altered cysteine metabolism and a deficiency of cysteine-containing antioxidants combined with a high capacity to oxidize fatty acids and generate ROS during aerobic exercise causes chronic oxidation and aggregation of key proteins such as desmin.

## BACKGROUND

Arabian horses are adept at endurance races, routinely competing in distances of 50–100 miles. Exertional rhabdomyolysis (ER), literally the dissolution of skeletal muscle with exercise, is a common occurrence in endurance horses, affecting 4–18% of competitors (68). While overexertion and environmental factors can cause ER, a genetic predisposition to ER is a common occurrence in some breeds (61). To date, however, genetic causes of ER have not been identified in the Arabian breed. We recently identified a muscle disease with a suspected genetic predisposition in Arabian horses called myofibrillar myopathy (MFM) that causes muscle stiffness and intermittent ER (45, 63).

Across species, MFM comprises a group of muscle diseases defined by morphological characteristics of Z-disc disarray, disruption of myofiber alignment, and ectopic accumulation of proteins that contain a variety of cytoskeletal and Z-disc material, depending upon the causative genetic mutation (56). The extent of ectopic protein aggregation, degree of change in mitochondrial staining, and severity of muscle weakness in equine MFM is frequently less than that described for human patients with MFM (53, 56, 63). Approximately half of all MFM cases in human medicine are caused by mutations in genes encoding sarcomeric and extrasarcomeric proteins, including desmin, filamin C, plectin, ZASP, myotilin, αB-crystallin, and BAG3, while the remaining diseases are due to still unresolved gene defects (20, 34) Although common in humans, Arabian horses with MFM do not appear to have impaired cardiac function as they have no clinical indicators of heart failure. Even when over 20 yr of age, MFM horses complete endurance rides without abnormal elevations in heart rates that are routinely evaluated at compulsory veterinary evaluations during and after endurance races (45). The etiopathology of MFM in horses and the protein(s) or gene(s) responsible remain unknown.

RNA-Seq and proteomic analyses have been of value in defining pathophysiological mechanisms for MFM and muscular dystrophies in human patients (24, 27). Three studies have evaluated the equine exercise transcriptome in healthy Thoroughbred or Arabian racehorses performing near-maximal to maximal exercise (7, 43, 52). To date, no studies have investigated the gluteal muscle equine transcriptome or proteome after aerobic exercise. We hypothesized that transcriptome and proteome analyses of skeletal muscle before and several hours after aerobic exercise would provide critical insights into the pathophysiology of MFM in horses.

## MATERIALS AND METHODS

### Aim

The objectives of the present study were to compare the skeletal muscle transcriptome and proteome of healthy and MFM affected Arabian horses at rest, to compare the alterations in gene expression that occurred with aerobic exercise and to compare the transcriptome of MFM and control horses 3 h after exercise. Procedures were approved by the Oregon State University Institutional Animal Care and Use Committee #4480, and horse owners provided informed consent.

### Horses

Horses for the study were recruited by contacting a large group of active endurance riders in the Pacific Northwestern US and selecting four Arabian and one Arabian-cross horse (age 15.8 ± 7.1 yr, 3 castrated males, 2 females) that had clear evidence of MFM based on clinical history of an exertional myopathy and histopathologic evaluation of muscle samples including desmin immunohistochemistry. Six control Arabian horses (13.0 ± 6.2 yr, 4 castrated males, 2 females) that were located on the same property and had similar feeding and exercise routines and no apparent histopathology in muscle biopsies were used as a matched control. All MFM horses had abnormal desmin-positive aggregates in gluteal muscle fibers (data reported previously) (63). ER was previously documented in 5 MFM horses, with the last episode at least 6 mo before taking part in the study. Control horses had participated in endurance activities for a minimum of 3 yr with no prior evidence of ER and had no evidence of muscle pathology in gluteal muscle biopsies. The muscle biopsy specimens utilized in the current experiment were aliquots of those obtained for previously published studies (45, 63).

### Standardized Exercise Test

An aerobic exercise test was used that all horses could complete regardless of their current fitness. The details of the test and the metabolic responses of the MFM and control horses included in the present study have been published previously (45). In brief, horses were stall rested for 24–48 h, and then a rider with a global positioning system-enabled watch guided the horse through a 47 min exercise test consisting of precise intervals of walk, trot, and canter over 6.4–7.2 km (4.0–4.5 miles). One owner of one control horse elected not to perform the exercise test, leaving five MFM and five control horses to complete the test with no differences in heart rate response, blood lactate, blood glucose, or plasma electrolyte concentrations identified between MFM and control horses (45). No horses displayed clinical signs of muscle stiffness during or after the exercise test. Plasma creatine kinase activities were within normal limits before and 3 h following exercise in all but one MFM horse that had a mild ∼2-fold elevation in creatine kinase activity (1116 U/l; normal range 145–633 U/l) following exercise (45).

### Muscle Samples

A 6 mm Bergstrom needle biopsy of the middle gluteal muscle was obtained at a depth of ~6 cm at a standardized site 17 cm along a line running from the dorsal tuber coxa to the tail head before and 3 h after exercise ceased on alternate sides of the gluteal muscle (63). Three hours postexercise was chosen because in a previous equine study, little differential expression (DE) of genes was noted immediately after exercise, whereas significant changes occurred 4 h after exercise (43). Samples for transcriptome analysis were immediately frozen in liquid nitrogen and stored at −80°C until analysis.

### Muscle Histochemistry

Samples for muscle histopathology obtained at rest were affixed to cork squares with optimal cutting temperature media and frozen in isopentane suspended in liquid nitrogen. Muscle fiber types were determined with myosin ATPase staining on 7 μm thick cryostat sections with 5 min preincubation at pH 4.4 (13). Muscle fiber type composition for type 1, 2A, and 2X fibers was determined by counting a minimum of 300 muscle fibers per horse. A one-way ANOVA and Tukey post hoc test was used to compare fiber type proportions between MFM and controls. Hematoxylin and eosin (HE) staining was performed on 7 μm thick cryostat sections.

### Immunohistochemistry

Immunohistochemistry was used to investigate desmin aggregation, regeneration, and myosin isoforms that had altered gene expression as well as peptide expression in the proteomic profile. Staining was performed for desmin (1:100 anti-desmin-mouse monoclonal D33; Agilent Technologies, Santa Clara CA) and MHCd (1:50 mouse monoclonal anti-MHCd; Leica Biosystems, Buffalo Grove, IL) as previously described (63, 64). For MYH13 staining, flash-frozen muscle samples 7 μm thick were placed on charged slides and air-dried overnight at 25°C. Endogenous enzyme blocking was performed in 0.3% hydrogen peroxide/Tris-buffered saline (TBS). After rinsing in tap water, pH adjustment was made with TBS, pH 7.4 (Scytek Laboratories, Logan, UT). Subsequently, standard micropolymer complex staining steps were performed at room temperature on the IntelliPath Flex Autostainer. All staining steps were followed by rinses in TBS Autowash buffer (Biocare Medical, Concord, CA). After blocking for nonspecific protein with Background Punisher for 10 min (Biocare), sections were incubated in rabbit polyclonal anti-MYH13 (PA5-70713; Thermo Fisher Scientific, Rockford, IL) diluted 1:50 in normal antibody diluent (Scytek). After rinsing, Promark Rabbit on Farma horseradish peroxidase micropolymer (Biocare) was applied for 30 min. Immunoreactivity was detected by using Romulin AEC for 5 min (Biocare). Slides were counterstained with CATHE hematoxylin.

### Transcriptomics

#### RNA extraction and sequencing.

Total muscle RNA was isolated with TRIzol/chloroform extraction and a biopulverizer (BioSpec Products, Fartlesville, OK) as previously described (22). DNase treatments were performed in columns (Direct-zol RNA MiniPrep Plus; Zymo, Irvine, CA) with TURBO DNase (Thermofisher, Wilmington, DE) according to manufacturer's instructions. Quantification and quality of RNA, along with degree of rRNA contamination, were assessed with the Pico chip on the Agilent Bioanalyzer 2100 (Santa Clara, CA), and samples with an RNA integrity number ≥7 were used for library preparation and quantification. Library construction with a strand-specific polyA^+^ capture protocol (TruSeq Stranded RNA, Illumina) was performed and sequencing completed using the Illumina HiSeq 2000 genome analyzer (100 bp PE) at a targeted 35 million reads/sample.

#### Mapping and assembling.

All paired end RNA-Seq reads were initially assessed for quality with FASTQC (1). Samples that passed through the quality threshold of 30 (Q ≥ 30) were aligned with the STAR aligner (16) to Ensembl horse CDS (EquCab2.86). A total of 26,993 sequences were used to create the index file and map RNA-Seq reads. The final number of reads aligned ranged from 31 to 38 M per sample, with uniquely mapped reads falling in the range of 87.92–94.12% (Supplemental Table S1). (The online version of this article contains supplemental material.) Count data for each sample were generated from the STAR-aligned BAM files using the internal flag in STAR. Differential gene expression (DE) was identified with EdgeR (51) generalized linear models (GLMs) available through R/Bioconductor (R Core Team, 2016), making assumptions to shrink gene-specific variance toward an underlying common variance for all genes (41, 51). The number of counts per million (CPM) were set to include a minimum of five horse samples. Pairwise differences among means and linear combinations of model parameters were used to evaluate the DE between control and MFM affected horses. The fixed effects model can be represented as: μ + αi + β_j +_ αβ_ij_, where μ is the overall mean, αi represents effects due to any confounding effects,β _j_ represents effects due to specific treatment, and αβ_ij_ represents interaction among effects. Such models are also flexible to incorporate nested effects, and specifically for these data we represent the model as: μ + α_treatment(horse)_, where μ is the overall mean, and α_treatment(horse)_ is the treatment effect nested within horse to accommodate repeated measures over each individual horse. There were no confounding covariates included in our model. All gene lists derived from pairwise comparisons were false discovery rate (FDR) corrected for multiple testing at a threshold of *P* < 0.05.

#### Enrichment and pathway analysis of transcriptomics.

Significant gene lists (*P* < 0.05) derived from pairwise comparison of transcriptomics data were analyzed with Cluego (2.3.2) (6), a Cytoscape (version 3.4.0) framework-based plug-in to visualize biological terms and large gene data sets and perform pathway analysis. The ENSEMBL gene IDs were mapped to the Gene Ontology (GO) database using all evidence without electronic annotation (IEA), which excludes computational annotations. Data were assessed at the biological level in GO. Default enrichment/depletion analysis was performed with a suitable background comprising ~11,000 genes, and *P* values were adjusted for multiple testing by Bonferroni step-down, which is the default in Cluego. The final list of genes evaluated in this study was derived from those genes that had enriched GO terms. Further information regarding the function of these genes was obtained from gencards.org.

### Transcriptome Profiling

After using the CPM feature to ensure that each transcript was present across at least five horses, we reduced the initial 26,993 transcripts identified in Ensembl to 11,330 transcripts, constituting the final transcripts used for identification of DE genes. Using the resulting list of genes from the final transcripts, we made pair-wise comparisons (pre- vs. postexercise, MFM vs. control at rest, MFM vs. control postexercise, MFM postexercise vs. control pre-exercise) with a GLM model. FDR-corrected significant genes (*P* < 0.05) were used for GO term analysis.

### Quantitative Real-time Polymerase Chain Reaction

Genes with >log_2_ fold higher or lower expression in MFM vs. control muscle were also evaluated by quantitative real-time polymerase chain reaction (qRT-PCR). Primer design is provided in Table 1. Thermocycling was conducted with EvaGreen dye (Biotium, Fremont, CA), ROX Reference Dye (Invitrogen, Life Technologies), and Hot Start Taq DNA Polymerase (New England BioLabs, Ipswich MA), using the QuantStudio 3 Real-Time PCR System (ThermoFisher Scientific, Rockford IL). PCR reactions were run in duplicate (20 µl volume reactions). Each reaction contained 2 µl of sample cDNA, 2 µl of 2.5 mM dNTPs, 2 µl of 10× PCR buffer, 1 µl of EvaGreen dye, 1.5 µl of 1:10 ROX Reference Dye dilution, 0.125 µl of Hot Start Taq DNA Polymerase, 2 µl of 1.6 µM forward primer, 2 µl of 1,600 µM reverse primer, and 7.4 µl of sterile nuclease free distilled water. Reactions were run for 40 cycles under the following conditions: denaturation at 95°C for 10 min, annealing at 60°C for 1 min; melt curve stages at 95°C for 15 s, 60°C for 1 min, and 95°C for 15 s. Cycle thresholds (CT) were automatically calculated by the QuantStudio 3 Real-Time PCR System. For each gene of interest 100% geometric efficiency was established. Nontemplate controls run for each gene showed no amplification. Mann-Whitney test was used to determine statistical significance with *P* < 0.05.

**Table 1.** Primers sets

Primer Set	Loci	Forward Primer (5′-3′)	Reverse Primer (5′-3′)	Annealing Temp., °C	Product Size, bp
ACTC1 (XM_023620570.1)	Exon 4–5	TGGTCGGGATCTCACTGACT	*ACGTTCAGCAGTGGTGACAA	57.5/57.1	76
DSP (XM_023624425.1)	Exon 6–7	*AAACCTGCTGAAAGCGTCCT	TCGCAGTCGTTGATCCACAT	57.3/56.7	102
USH2A (XM_001915645.3)	Exon 1–2	TTCTTCTCCTCCTTCTCCAACG	*GCCGTCTTTTCTATAACACACATTA	56.2/53.3	99
DLK1 (XM_001917562.3)	Exon 3–4	GGACTCTGTGTGGAACCCTG	*CAGGCCCGGATGTCTAAGTC	57.6/57.4	80
CBS (XM_023630242.1)	Exon 10–11	CCGACTCGGTTCGCAACTA	CCTCCTCATTCAGGAAGCC	59.8/57.2	78
GAPDH (NM_001163856.1)	Exon 4–5	ATGATTCCACCCATGGCAAGT	*GGATCTCGCTCCTGGAAGATG	59	100

Primers sets used for quantitative RT-PCR sequencing of cardiac alpha actin (*ACTC1*), desmoplakin (*DSP*), Usherin (*USH2A*), delta notch-like protein 1 (*DLK1*), cystathione beta synthase (*CBS*), and housekeeping gene glyceraldehyde 3 phosphate dehydrogenase (*GAPDH*). *Whether exon junction was on the forward or reverse primer.

### Coding Sequence of Desmin

Potential mutations in the coding sequences of desmin were investigated as a putative cause of MFM by using initial raw fastq files realigned using the STAR two-pass alignment method (13), and the resulting BAM files were sorted; duplicates were marked (http://broadinstitute.github.io/picard/), realigned, and recalibrated with GATK (44). The resulting BAM files were then used as an input to call variants with SAMtools (38). The resulting VCF file was filtered by BCFtools (46), and a final list of single nucleotide polymorphisms (SNPs) was compared with a case vs. control setup using SnpSift (10). The resulting SNP data were also annotated for variant function (synonymous or nonsynonymous).

### Proteomics

#### Sample preparation, peptide fractionation, and mass spectrometry.

We reconstituted 20 mg samples of frozen ground tissue from the same gluteal muscle biopsy obtained at rest from a subset of horses used for RNA-Seq (3 MFM mean age 20 yr, 3 castrated males and 3 control mean age 17 yr, 3 castrated males) with a ratio of 10 μl extraction buffer [7 M urea, 2 M thiourea, 0.4 M triethylammonium bicarbonate (TEAB) pH 8.5, 20% acetonitrile and 4 mM Tris (2-carboxyethyl) phosphine] per mg of tissue, while on ice. The samples were vortexed briefly and then sonicated at 30% amplitude for 7 s in a Branson Digital Sonifier 250 (Branson Ultrasonics, Danbury, CT). For each sample, 150 μl was transferred to a PCT tube (Pressure Cycling Technology; Pressure Biosciences, S. Easton, MA) with a 150 μl cap for the Barocycler NEP2320 (Pressure Biosciences) and cycled between 35 kpsi for 30 s and 0 kpsi for 15 s for 40 cycles at 37°C. The PCT tube was uncapped, and 200 mM methyl methanethiosulfonate (MMTS) was added to a final concentration of 8 mM MMTS, recapped, inverted several times, and incubated 15 min at room temperature. The samples were transferred to a new 1.5 ml microfuge Eppendorf Protein LoBind tube. Two aliquots for each sample were taken for protein concentration determination by Bradford assay.

For in-solution proteolytic digestion and isobaric tags for relative and absolute quantitation (iTRAQ) labeling, a 100 μg aliquot of each sample was transferred to a new 1.5 ml microfuge tube and brought to the same volume with protein extraction buffer and 8 mM MMTS. All samples were diluted fourfold with ultrapure water and trypsin (Promega, Madison, WI), added in a 1:35 ratio of trypsin to total protein. Samples were incubated overnight for 16 h at 37°C after which they were frozen at −80°C for 30 min and dried in a vacuum centrifuge. Each sample was then cleaned with a 4 ml Extract Clean C18 SPE cartridge from Grace-Davidson (Deerfield, IL); eluates were vacuum dried and suspended in dissolution buffer (0.5 M TEAB, pH 8.5) to a final 2 μg/μl concentration. We labeled 40 μg for each sample with iTRAQ 8-plex reagent (AB Sciex, Foster City, CA) per manufacturer’s protocol. After labeling, the 40 μg aliquot of each sample was multiplexed together into one 1.5 ml tube and vacuum-dried. The multiplexed sample was cleaned with a 3 CC Oasis MCX SPE cartridge (Waters, Milford, MA), and the eluate was dried in vacuo.

The iTRAQ-labeled samples were resuspended in *buffer A* (10 mM ammonium formate pH 10 in 98:2 water-acetonitrile) and fractionated offline by high-pH C18 reversed-phase (RP) chromatography (69). A MAGIC 2002 HPLC (Michrom BioResources, Auburn, CA) was used with a C18 Gemini-NX column, 150 mm × 2 mm internal diameter, 5 μM particle, 110 Å pore size (Phenomenex, Torrence, CA). *Buffer A* was 10 mM ammonium formate, pH 10 in 98:2 water-acetonitrile, and *buffer B* was 10 mM ammonium formate, pH 10 in 10:90 water-acetonitrile. The flow rate was 200 μl/min with a gradient from 5 to 35% *buffer B* over 60 min, followed by 35–60% over 5 min. Fractions were collected every 2 min, and UV absorbances were monitored at 215 and 280 nm. Peptide containing fractions were divided into two equal numbered groups: “early” and “late.” The first early fraction was concatenated with the first late fraction, and so on. Concatenated samples were dried in vacuo, resuspended in loading solvent (98:2:0.01, water-acetonitrile-formic acid), and 1–1.5 µg aliquots were run on a Velos Orbitrap mass spectrometer (Thermo Fisher Scientific, Waltham, MA) as described previously (39) with the following modifications: lock mass was not used, HCD activation time was 20 ms, dynamic exclusion duration was 15 s, and the minimum signal threshold for data-dependent trigger was 20,000 counts. The mass spectrometer RAW data were converted to mzXML using MS Convert software from the ProteoWizard Toolkit (PMID: 23051804) and to MGF files using TINT raw-to-mgf converter (in-house tool).

#### Protein identification and statistical analysis.

MGF-formatted files were used for identification of peaks by using several search engines to limit potential pitfalls of using a single algorithm/method and to perform more exhaustive survey of the spectra (57). Multiple search engines such as OMSSA (version 2.1.9) (25), X!Tandem Vengeance (2015.12.15.2) (12), Andromeda (version 1.5.3.4) (11), MS Amanda (version 1.0.0.7501) (17), MS-GF+ (version Beta v10282) (33), Comet (version 2016.01 rev. 2) (19), MyriMatch (version 2.2.140) (58), and Tide (15) were invoked within the SearchGUI (version 3.2.12) framework (65), and results collated with PeptideShaker (version 1.16.4) (65).

Protein identification was conducted against a concatenated target/decoy (18) version of the *Equus caballus* (28,232, >99.9%), *Equus ferus caballus* (1, < 0.1%), *Sus scrofa* (1, < 0.1%) complement of the UniProtKB (version October, 2016) (2), with 28,234 (target sequences). The decoy sequences were created by reversing the target sequences in SearchGUI. The identification settings were as follows: *1*) trypsin, specific cleavage, with a maximum of two missed cleavages 10.0 ppm as MS1 and 0.05 Da as MS2 tolerances; *2*) fixed modifications: carbamidomethylation of C (+57.021464 Da); *3*) iTRAQ specific modifications 8-plex of peptide N-term (+304.199039 Da) and iTRAQ 8-plex of K (+304.199039 Da); and *4*) variable modifications: oxidation of M (+15.994915 Da), iTRAQ 8-plex of Y (+304.199039 Da).

Peptides and proteins were inferred from the spectrum identification results with PeptideShaker (version 1.16.4) (65). Peptide spectrum matches (PSMs), peptides, and proteins were validated at a 1.0% FDR estimated from the decoy-hit distribution. The resulting file was imported into Reporter package (version 0.7.2) (http://compomics.github.io/projects/reporter.html), which was included as complementary tool within the SearchGUI platform. Peptides that could belong to different members of the same protein family were handled by using stringent filtering and the Occam's razor approach. The three rules used by this approach were *1*) For two proteins to be clustered, the sum of the probabilities of their shared peptides must be at least 95%. *2*) The proteins must share at least 50% of their evidence. This is determined by summing the probabilities of the shared peptides and comparing this value with the summed probabilities of all of the peptides for each individual protein. If the sum of the probabilities of the shared peptides is greater than or equal to half of the sum of the peptide probabilities for either of the individual proteins, a cluster is formed. *3*) A protein may be included in an existing cluster if it meets the above criteria with a member protein of the cluster. For two proteins to be clustered in a group, the sum of the probabilities of their shared peptides must be at least 95%.

To normalize data to controls, ratios were created per protein by dividing mean MFM values by mean control values. The resulting data table was exported as a text file, log transformed, and analyzed to determine significance between treatments with Perseus (62). *P* values were adjusted for multiple testing by the permutation-based FDR method included within Perseus. Individual spectra for all proteins with >log_2_ fold change were manually checked for each sample/animal to ensure there were no missing data. The biological significance of the final list of confident proteins was assessed with the Panther protein classification tool (59).

### Transcriptomic vs. Proteomic Analyses

To find intersecting IDs between transcriptomics and proteomic data sets, we first converted all confident proteins (673 protein IDs) expressed in MFM and control horses to gene names with the ENSEMBL BioMart resource. Significant gene IDs (*P* < 0.05) from MFM vs. Control transcriptomics at rest (284 genes) and MFM vs. Control transcriptomics after exercise (191 genes) were evaluated for genes that had overlap with Gene IDs identified from confident proteins in the proteomics data. Because long-term changes in gene expression are required to alter protein expression, proteomic analysis was performed only on at-rest and not on postexercise samples (24).

## RESULTS

### Muscle Fiber Type Composition and Histopathology

**Fig. 1.**
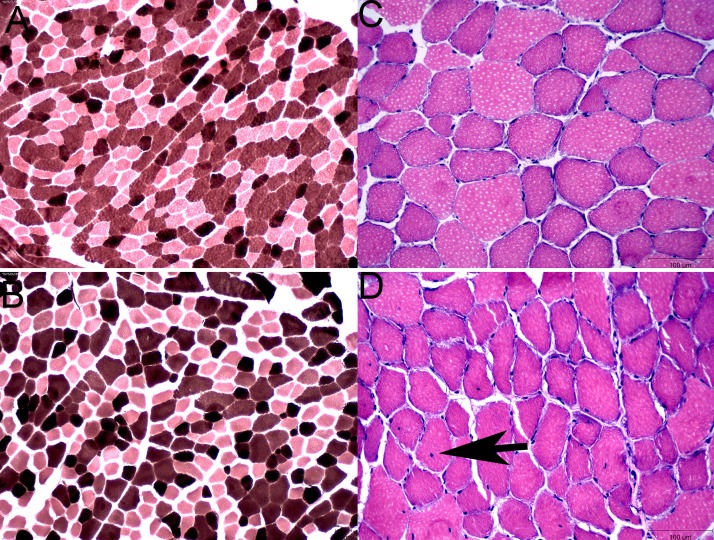
*A*: ATPase stain of a control horse showing a mosaic pattern of type 1 (black), 2A (light), and 2X (brown) fiber types. Myosin ATPase pH 4.4 10×. *B*: ATPase stain of an MFM horse showing the same mosaic pattern and fiber type distribution as the control. Myosin ATPase pH 4.4 10×. *C*: normal gluteal muscle of a control horse. 20× HE. *D*: gluteal muscle from a horse with MFM showing internalized myonuclei (arrow) and slight increase in endomysial connective tissue. 20× HE. HE, hematoxylin-eosin; MFM, myofibrillar myopathy.

There was no significant difference in skeletal muscle fiber type composition between MFM and control horses (Fig. 1, *A* and *B*). Fiber type composition was type 1 (MFM 17 ± 3%; control 17 ± 3%), type 2A (MFM 56 ± 7%; control 52 ± 8%), and type 2X fibers (MFM 27 ± 4%; control 31 ± 8%). Neither acute myofiber necrosis nor macrophage infiltration was evident in HE stains. Mature myofibers with small internalized myonuclei were present in 5/5 MFM horses and in 2/6 control horses. Increased endomysial or perimysial connective tissue was not evident in all MFM horses; however, two of the most severely affected MFM horses appeared to have a slight increase in endomysial connective tissue in some regions (Fig. 1, *C* and *D*).

#### Immunohistochemistry.

**Fig. 2.**
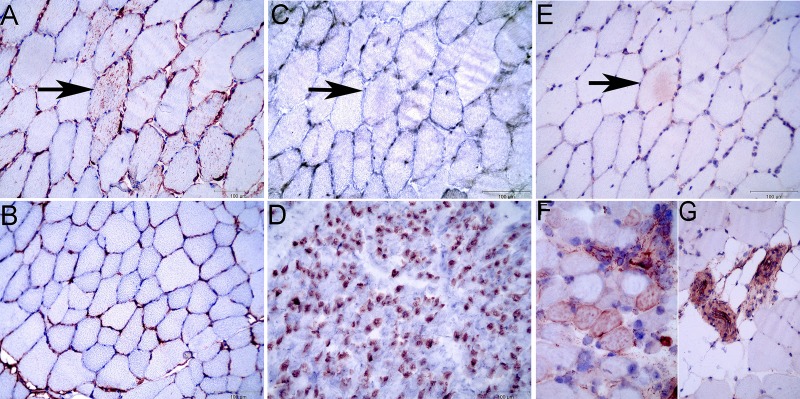
*A*: desmin staining of an MFM horse showing aggregates of desmin in numerous fibers. Desmin immunohistochemistry (IHC) 20×. *B*: control gluteal muscle stained for desmin. Desmin IHC 20×. *C*: serial section to *A* stained for developmental myosin showing complete lack of regenerative fibers. Arrow indicates same fiber as in *A*. MyoD IHC 20×. *D*: fetal horse muscle stained for developmental myosin MyoD IHC 40×. *E*: serial section to *A* stained for extraocular myosin showing faint staining in only one desmin-positive fiber (arrow). MYH13 IHC 20×. *F*: extraocular muscle fibers showing MYH13 of extraocular muscle fibers and blood vessels. MYH13 IHC 40×. *G*: blood vessels in gluteal muscle of a control horse staining positive for MYH13. MYH13 IHC 20×.

Immunohistochemistry identified desmin-positive aggregates in scattered myofibers of MFM but not control horses (Fig. 2, *A* and *B*). Neither small basophilic fibers with centrally located nuclei nor fibers staining positively for developmental myosin MYHd typical of regeneration were observed in any horses (Fig. 2, *C* and *D*). As a validation step for proteomic expression, immunohistochemical staining was performed for MYH13. Positive staining for MYH13 was found in control samples of extraocular muscle fibers and was also found in the walls of small blood vessels in MFM and control samples (Fig. 2, *E*–*G*). Only one desmin-positive skeletal muscle fiber in one MFM horse had mild focal MYH13 staining (Fig. 2*E*). Expression of MYH13 in proteomic data could reflect overlap in amino acid sequence with vascular smooth muscle myosin.

### Transcriptomics

#### Control horses: effect of aerobic exercise.

**Fig. 3.**
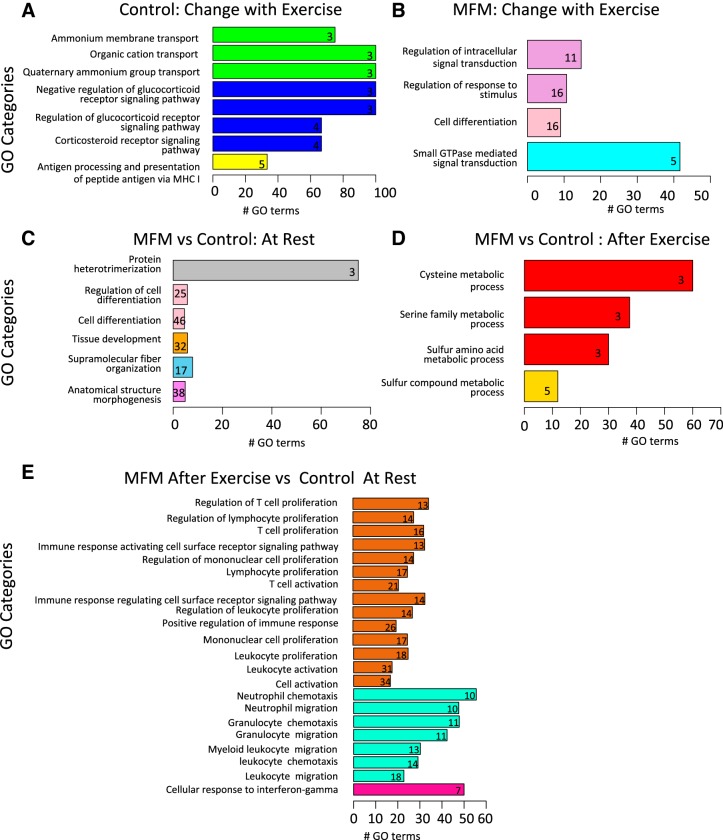
Gene Ontology (GO) term analysis for enriched biological pathways of differentially expressed (DE) genes obtained through pair-wise comparison between each treatment. *y*-axis represents GO categories identified in the GO analysis. Similar GO categories are identified by similar colors. The *x*-axis represents the number of GO terms. Numbers within bar plots represent the number of GO terms significantly (*P* > 0.05) associated with the enriched pathway for each biological function. *A*: glucocorticoid signaling (blue) and cation transport (green) were primarily modified by exercise in control horses (pre- vs. postexercise). *B*: unlike control horses, signal transduction and cell differentiation were primarily modified in MFM horses (pre- vs. postexercise). *C*: pathways involved in anatomic structure, fiber organization, and cell differentiation were primarily different in MFM vs. control horses at rest. *D*: after exercise, pathways exclusively involving sulfur containing compounds that impact cysteine metabolism were different between MFM vs. control horses 3 h after exercise. *E*: GO pathways involved in inflammation or the immune system were primarily different when comparing muscle from MFM horses after exercise to resting control muscle.

The comparison of transcriptomes of control horses at rest vs. after aerobic exercise identified 330 DE genes (adj. *P* < 0.05) (Supplemental Table S1). After GO enrichment analysis, 12 genes had significant GO terms in biological processes involving corticosteroid receptor pathways (GO:0031958, GO:0042921, GO:2000322, GO:2000323), organic cation/quaternary ammonium transport (GO:0015695, GO:0015697, GO:0015101, GO:0072488, GO:0015651), and antigen processing via major histocompatibility class 1 (GO:0002474) (Fig. 3*A*). With an additional stringent threshold of >1.0 log_2_ fold change applied to these 330 DE genes, the aryl hydrocarbon receptor nuclear translocator-like protein 1 (*ARNTL* ↓2.50 MFM), a clock gene involved in circadian rhythm, and an organic cation solute carrier (*SLC22A3* ↓1.76 MFM) were identified as downregulated, and a clock gene (*PER1* ↑1.32 MFM) was upregulated (Table 2).

**Table 2.** Genes significantly differentially expressed

Gene Symbol	Gene Name	GO Term	Log_2_ Fold		Adj. *P* Value
*A. MFM vs. control at rest*					
*ACTC1*	actin, alpha, cardiac muscle 1	supramolecular fiber organization	↑	2.47	1.05E-06
*DSP*	desmoplakin	supramolecular fiber organization	↑	2.38	8.89E-24
*MYOZ2*	myozenin 2	supramolecular fiber organization	↓	1.03	1.82E-13
*FREM2*	FRAS1 related extracellular matrix protein 2	extracellular matrix component	↑	1.17	1.36E-06
*USH2A*	usherin	extracellular matrix component	↓	2.93	1.48E-10
*PITX3*	paired like homeodomain 3	tissue development/transcription	↑	1.01	7.70E-04
*FEZ1*	fasciculation and elongation protein zeta 1	cell differentiation/axonal growth	↑	1.19	5.06E-05
*PLCG2*	phospholipase C gamma 2	cell differentiation/inflammation	↑	1.01	6.72E-06
*DLK1*	delta like noncanonical Notch ligand 1	calcium ion binding	↓	2.39	1.36E-04
*ASTN2*	astrotactin 2	calcium ion binding	↓	1.40	4.83E-15
*B. MFM vs. control after exercise*					
*CBS*	cystathionine-beta-synthase	cysteine metabolic process	↓	2.17	1.93E-09
*SLC7A10*	solute carrier family 7 member 10	cysteine membrane transport	↓	1.80	4.08E-07
*SLC24A1*	cationic amino acid transporter 4-like	amino acid membrane transport	↓	1.11	4.38E-05
*C. MFM Before vs. after exercise*					
*GADD45G*	growth arrest and DNA damage inducible gamma	regulation of response to stimulus	↑	1.68	1.53E-04
*ARNTL*	aryl hydrocarbon receptor nuclear translocator-like protein 1	regulation of response to stimulus	↓	2.18	7.64E-10
*RCAN1*	regulator of calcineurin 1	regulation of response to stimulus	↓	1.07	2.62E-06
*ARHGAP30*	Rho GTPase activating protein 30	small GTPase mediated signaling	↓	1.39	4.91E-05
*SYK*	spleen-associated tyrosine kinase	cell differentiation	↓	1.36	1.27E-02
*SPI1*	Spi-1 proto-oncogene	cell differentiation	↓	1.05	6.28E-03
*D. Control before vs. after exercise*					
*PER1*	period circadian clock 1	corticosteroid receptor signaling (circadian rhythm)	↑	1.31	1.14E-05
*ARNTL*	aryl hydrocarbon receptor nuclear translocator-like protein 1	corticosteroid receptor signaling (circadian rhythm)	↓	2.50	3.33E-15

Genes significantly differentially expressed with RNA-Seq after Gene Ontology (GO) term analysis >1.0 log_2_ fold change in horses at rest (*A*), after exercise (*B*), and comparing rest with after exercise (*C, D*). MFM, myofibrillar myopathy.

#### MFM horses: effect of aerobic exercise.

The comparison of MFM transcriptomes at rest vs. postexercise identified 349 DE genes (adj. *P* < 0.05) (Supplemental Table S1). These genes had different GO terms compared with rest vs. after exercise in control horses (Fig. 3*B*). After enrichment analysis, 27 genes had significant GO terms in biological processes that involved regulation of intracellular signal transduction (GO1902531) and response to stimulus (GO:0048583), small GTPase signaling (GO:0007264), and cell differentiation (GO:0030154) (Fig. 3*B*). When the additional stringent filter of >1.0 log_2_ fold expression was applied, one gene had higher expression after exercise within signaling, growth arrest, and DNA damage inducible gamma (*GADD45G*, ↑1.68 MFM), and five genes involved in signaling or cell differentiation had less expression with exercise *ARNTL* (↓2.18 MFM), Rho GTPase activating protein (*ARHGAP30* ↓1.39 MFM), spleen-associated tyrosine kinase (*SKY* ↓1.37 MFM), regulator of calcineurin 1 (*RCAN1* ↓1.07), and Spi-1 proto-oncogene (*SPI1* ↓1.05 MFM) (Table 2).

### MFM vs. Control Horses at Rest

We found 284 genes showing significant DE (adj. *P* < 0.05) between MFM and control horses at rest (Supplemental Table S1). After enrichment analysis, 62 genes had significant GO terms for biological processes involving structural morphogenesis (GO:0009653), muscle fiber organization (GO:0097435), tissue development (GO:0009888), cell differentiation (GO:0030154, GO:0045595), and protein heterotrimerization (GO:0070208) (Fig. 3*C*). Expression of four genes was altered >1.0 log_2_ fold in MFM vs. control horses (Table 2); cardiac alpha actin (*ACTC1*, ↑2.47 MFM) and myozenin 2 (*MYOZ2* ↓1.03 MFM) in the sarcomere; desmoplakin (*DSP*, ↑2.3 MFM) in the cytoskeleton; usherin (*USH2A*, ↓2.93 MFM), and *FRAS1*-related extracellular matrix protein 2 (*FREM2* ↑1.17 MFM) in the basement membrane/extracellular matrix; paired like homeodomain 3 (PITX3 ↑1.02 MFM), phospholipase C gamma 2 (PLCG2 ↑1.00 MFM), and fasciculation and elongation protein zeta 1 (FEZ1 ↑1.19 MFM) involved in cell differentiation and delta like noncanonical Notch ligand 1 (*DLK1*, ↓2.39 MFM) and astrotactin (ASTN2 ↓1.40 MFM) involved in calcium ion binding (Table 2).

### MFM vs. Control Horses Following Aerobic Exercise

We found 191 genes showing significant DE (adj. *P* < 0.05) in muscle of MFM compared with control horses following exercise (Supplemental Table S1). After enrichment analysis, there were nine genes with significant GO terms that involved amino acid transport (GO:0015179, GO:0015297) and sulfur compound or sulfur amino acid metabolic processes (methionine, cysteine, taurine) (GO:0006790 GO:0000096, GO:0009069, GO:0006534) (Fig. 3*D*). Three genes had >1.0 log_2_ fold DE in MFM vs. control horses: cystathionine-beta-synthase (*CBS*, ↓4.51 MFM) involved in cysteine synthesis and two membrane transporters, one for cysteine and neutral amino acids (*SLC7A10*, ↓1.79 MFM) and the other a potassium-dependent sodium/calcium exchanger (*SLC24A1*, ↓2.16 MFM) (Table 2, Fig. 3*B*).

### MFM Horses Postexercise vs. Resting Control Horses

This analysis was performed to compare the extremes: DE expression after exercise in MFM horses to a healthy resting horse. Significant DE of 883 genes was found when comparing MFM muscle after exercise to resting control muscle. This encompassed 305 genes with 22 significant GO terms (Table 3), many involving inflammation/immune response and cell activation (Fig. 3*E*). Four sarcomeric genes had >1.0 log_2_ higher expression in MFM (*ACTC1*, ↑2.75 MFM): ankrin repeat domain 1 (*ANKRD1*, ↑1.50 MFM), a myofibrillar stretch sensor and regulator of lipid metabolism; myosin heavy chain 13 (*MYH13* ↑1.63 MFM, *P* = 1.17E-33); myosin heavy chain 3 (*MYH3* ↑1.27 MFM, *P* = 1.19E-06); as well as cytoskeletal gene *DSP* (↑1.36 MFM). Other genes with >1.0 log_2_ altered expression that overlapped with those DE in comparisons of MFM vs. control at rest or after exercise included *GADD45G* (↓1.57 MFM), *ASTN2* (↓1.50 MFM), *PER1* (↓1.67 MFM), *ARHGAP30* (↑1.34 MFM), *SYK* (↑1.16 MFM), and *SPI1* (↑1.17 MFM) (Supplemental Table S1).

**Table 3.** Significant GO IDs for enriched biological pathways of DE genes comparing postexercise MFM muscle with resting control muscle

GO Term Biological Process	GO ID	DE Genes, *n*
Cellular response to interferon-gamma	GO:0071346	7
Cell activation	GO:0001775	34
Positive regulation of immune response	GO:0050778	26
Leukocyte migration	GO:0050900	18
Leukocyte chemotaxis	GO:0030595	14
Myeloid leukocyte migration	GO:0097529	13
Leukocyte activation	GO:0045321	31
Leukocyte proliferation	GO:0070661	18
Regulation of leukocyte proliferation	GO:0070663	14
Regulation of lymphocyte proliferation	GO:0050670	14
Lymphocyte proliferation	GO:0046651	17
Regulation of T cell proliferation	GO:0042129	13
T cell proliferation	GO:0042098	16
T cell activation	GO:0042110	21
Regulation of mononuclear cell proliferation	GO:0032944	14
Mononuclear cell proliferation	GO:0032943	17
Granulocyte migration	GO:0097530	11
Granulocyte chemotaxis	GO:0071621	11
Neutrophil migration	GO:1990266	10
Neutrophil chemotaxis	GO:0030593	10
Immune response-regulating cell surface receptor signaling pathway	GO:0002768	14
Immune response-activating cell surface receptor signaling pathway	GO:0002429	13

DE, differentially expressed.

### qRT-PCR Validation of DE Transcripts

The transcripts with >2 log_2_ fold change in expression in resting RNA-Seq data (MFM vs. control) included *ACTC1* (↑log_2_ FC 2.5), *DSP* (↑log_2_ FC 2.4), *DLK1* (↓log_2_ FC 2.4), and *USH2A* (↓log_2_ fold 2.9). A similar pattern of increased or decreased expression was found for these genes by qRT-PCR (Table 4). A significant increase in expression was found for *DLK1* (*P* = 0.004), and *USH2A* approached statistical significance (*P* = 0.052) with the small number of samples in the comparison (5 MFM vs. 6 control). Additionally, lower CBS expression in RNA-Seq data for MFM vs. control after exercise (↓log_2 FC_ 4.5) was also found with qRT-PCR, and this difference approached statistical significance (*P* = 0.056) (Table 4).

**Table 4.** Quantitative real-time PCR validation of specific transcripts from RNA-Seq

Gene	dCT MFM	dCT Control	2^-ddCT^	*P* Value
*MFM vs. control at rest*				
*ACTC1*	8.02	9.41	2.62	0.178
*DSP*	12.88	13.77	1.85	0.662
*USH2A*	11.82	9.15	0.16	0.052
*DLK1*	13.81	9.65	0.06	0.004
*MFM vs. control after exercise*				
*CBS*	14.81	12.82	0.25	0.056

Statistical significance was determined with Mann-Whitney test.

### Desmin Coding Sequence from RNA-Seq

Thirty-eight variants existed in the coding sequence of desmin between MFM and control horses; however, not one variant was consistently present in each MFM horse and consistently absent in control horses. There was one synonymous variant (Asp89Asp) that was present in five MFM as well as five control horses. The other identified variants were intronic (*n* = 37), of which nine were potential splice region variants that did not segregate exclusively in MFM horses.

### Proteomics

#### Confident protein identification.

Using the target/decoy strategy on MFM and Control samples, we found 28,232 proteins to match our proteome spectra. SearchGUI identified 11,876 of these proteins with coverages ranging from >0.19 to 100%. The default setup of FDR (1%) and FNR (<5%) resulted in 990 proteins passing threshold. We identified 21 proteins that had duplicate ID; however, they were not included in further analyses because none of them passed threshold. There were no missing data with significant proteins. A further filtering of proteins in Peptide Shaker using default confidence scores resulted in a final list of 673 proteins that were classified as “confident.”

#### Protein classification.

**Fig. 4.**
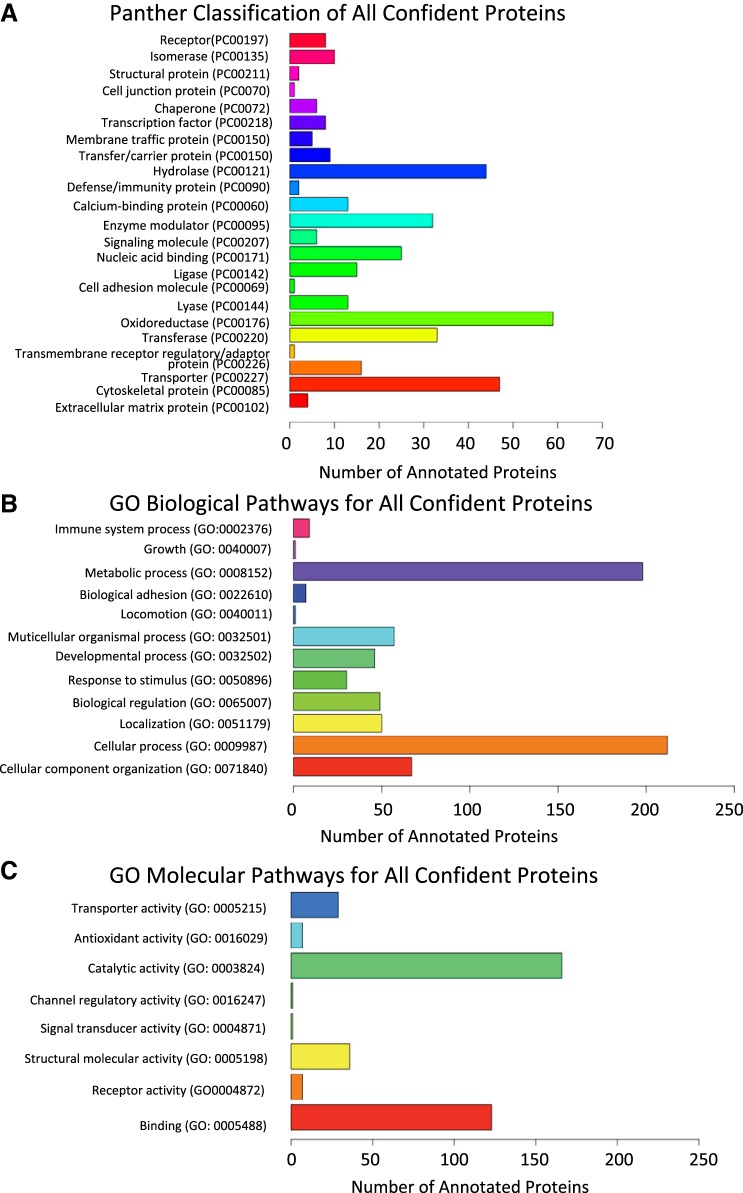
Classification of pathways for the 673 confident proteins in MFM and control horse muscle obtained at rest. Proteins used in the analysis were identified by a Search GUI protein identification protocol and had UniProtKB IDs. *A*: the largest number of proteins in equine muscle were classified as oxidoreductase, hydrolase, or cytoskeletal proteins by Panther analysis using Protein Plus tools. *B*: metabolic and cellular processes were the two major biological pathways for proteins in equine muscle according to GO biological pathway classification. *C*: catalytic activity and binding were the two major molecular pathways for proteins in equine muscle according to GO molecular pathway classification.

The 673 confident proteins were classified by the Panther protein class tool into 23 different protein classes of which oxidoreductase, cytoskeletal proteins, and hydrolase had over 40 incorporated proteins each (Fig. 4*A*) (60). Within this classification, there were 101 mitochondrial proteins (24 were Complex I) and 15 oxidoreductase proteins, including one catalase, four PRDX (1, 2, 5, 6), two thioredoxin, four glutathione, and four superoxide dismutase proteins. GO Biological pathway analysis of the confident proteins identified two primary biological pathways that involved either metabolic or cellular processes each containing >175 proteins. There were 10 other biological pathways that contained 75 or fewer proteins in MFM and control horse muscle (Fig. 4*B*). GO Molecular pathway analysis identified two major molecular pathways that involved catalytic activity or binding activity that contained >100 proteins and six other pathways that contained <40 proteins each (Fig. 4*C*).

#### DE of proteins.

**Fig. 5.**
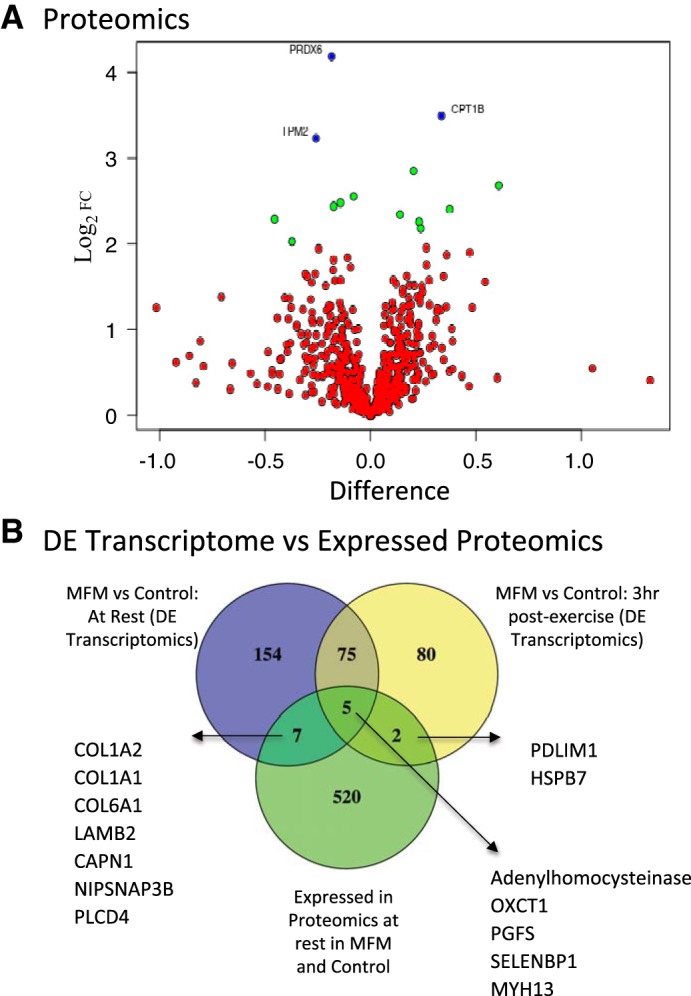
*A*: volcano plot of iTRAQ proteomic analysis illustrating the false discovery rate (FDR) corrected, statistically significantly altered proteins (blue dots) in MFM vs. control horse muscle at rest (3 MFM vs. 3 control horses). Proteins with >2 fold log_2_ change (FDR corrected *P* < 0.2) are shown as green dots and listed in Table 5. *B*: Venn diagram showing overlap among confident proteins (UniProtKB IDs with Gene IDs) in MFM and control muscle at rest and significantly DE genes after GO enrichment in MFM vs. control horses either at rest or 3 h after exercise. Numbers indicate the number of overlapping genes within each sector, tables provide a list of DE genes that overlap with identified proteins.

Statistical analysis identified three significantly DE proteins between MFM and control horses after controlling for multiple comparisons. The individual spectra were manually checked for each sample/animal to ensure there were no missing data for these three proteins. The three proteins were an antioxidant peroxiredoxin 6 (PRDX6, ↓log_2_ 4.14 MFM); an enzyme that transports fatty acids across mitochondrial membranes, carnitine palmitoyl transferase (CPT1B ↑log_2_ 3.49 MFM); and sarcomeric protein tropomyosin beta chain (TPM2 ↓log_2_ 3.24 MFM) (Fig. 5*A*, Table 5). Biological processes were evaluated through the additional inclusion of proteins with >2 log_2_ fold change (11 proteins, adjusted *P* values between *P* = 0.113 and 0.182) (Fig. 5*A*, Table 5). The major processes included thiol-specific antioxidants, oxidative metabolism, supramolecular fiber organization, proteasome degradation, apoptosis, immune function, and ribosomal/purine synthesis.

**Table 5.** Proteins that were differentially expressed at >2 log2 fold in MFM compared with control horses at rest

Protein Group	Gene Symbol	Gene Name	Process	Log_2_ FC		Adj. *P* Value
F7AXI9	*PRDX6*	peroxiredoxin-6	thiol-specific antioxidant	↓	4.14	0.0001*
F6S972	*CPT1B*	carnitine palmitoyltransferase 1	oxidative/fat metabolism	↑	3.49	0.0001*
F6WY50	*NDUFS4*	NADH dehydrogenase iron-sulfur protein 4	oxidative metabolism	↓	2.64	0.133
K9KEN6	*NDUFA5*	NADH dehydrogenase 1 alpha subcomplex subunit 5	oxidative metabolism	↓	2.44	0.115
F7AY21	*UNC45B*	protein unc-45 homolog B	sarcomere formation/myosin	↑	2.27	0.121
F7A281	*TPM2*	tropomyosin beta chain	sarcomere structure/actin	↓	3.24	0.039*
F6ZRJ3	*TCAP*	telethonin	sarcomere structure/actin	↓	2.49	0.114
F6ZTR4	*TPM3*	tropomyosin alpha-3 chain	sarcomere structure/actin	↓	2.03	0.182
F6TY28, F6UUU2	*UTRN*	utropin	sarcolemma/basement membrane	↑	2.17	0.130
D2KAT3	*PSMC3*	26S protease regulatory subunit 6A	proteosome/peroxisome	↑	2.40	0.111
F7D7B2	*DIABLO*	Diablo homolog	apoptosis	↑	2.85	0.12
F6Y6W2	*RPL24*	60S ribosomal protein L24	ribosomal/purine synthesis	↓	2.68	0.130
F7DBJ7	*RPS7*	40S ribosomal protein S7	ribosomal/purine synthesis	↓	2.37	0.111
Q95M34	*IGHC1*	immunoglobulin gamma 1 heavy chain constant region	immune system	↑	2.29	0.112

*Significantly DE proteins.

### Overlap between Significantly DE Genes and Identified Proteins

Out of the total 673 confident proteins, 543 had encoding gene IDs present in both transcriptomic and proteomic data sets. These confident proteins were identified but not DE in MFM and control horses. When comparing the proteomic data set with DE genes in resting MFM vs. control muscle, we found seven significantly DE genes that had proteins identified by proteomic analysis (Fig. 5*B*). These included adenylhomocysteinase (LOC100054381), two proteins involved in ketone body metabolism (OXCT1, PGFS), and extraocular muscle myosin MYH13.

When we compared DE genes in postexercise MFM vs. control muscle with all the identified proteins in the resting muscle proteomic data set, two proteins, PDLIM1 (cytoskeletal protein) and HSPB7, which act to prevent protein misfolding, overlapped with the DE genes (Fig. 5*B*). There were five proteins identified in the proteomic data set of MFM and control horses that had encoding gene transcripts DE in MFM vs. control both at rest and after exercise (Fig. 5*B*). These included a cysteine endopeptidase (CAPN1), phospholipase C (PLCD4), a vesicle transport protein (NIPSNAP3B), and four proteins involved in the extracellular matrix (COL1A1, COL1A2, COL6A1, LAMB2). None of these five proteins were DE in MFM horses, and none of the DE genes had >1 log_2_ fold DE.

### Availability of Data

The data sets generated and/or analyzed during the current study are available in the National Center for Biotechnology Information's Gene Expression Omnibus (GEO) (17a) and are accessible through GEO Series accession number GSE104388. The mass spectrometry proteomics data have been deposited to the ProteomeXchange Consortium via the PRIDE (66) partner repository with the data set identifier “PXD009362”.

## DISCUSSION

The present study represents the first study of the combined proteome and exercise transcriptome in horses and the first to utilize these analyses to probe muscle disease pathogenesis in horses. MFM Arabian horses showed DE of genes and proteins involved in pathways of sarcomere and cytoskeletal structure, oxidoreductase activity, fatty acid and amino acid metabolism, proteosomal degradation, and apoptosis. These alterations in expression do not appear to reflect a general myopathic process, because microarray studies of equine polysaccharide storage myopathy (PSSM1) and equine recurrent exertional rhabdomyolysis (RER) do not show overlap with DE genes in the present study, particularly when evaluating the top 10 DE genes in these studies (4, 5). Future RNA-Seq and proteomic analyses of other equine myopathies will help to elucidate which specific proteins and genes are DE as secondary effects of muscle disease and which are specific to MFM in Arabian horses.

Increases in connective tissue and muscle fiber regeneration are common features of many myopathies. It was interesting to note that four collagen and laminin proteins involved in the extracellular matrix had expressed peptides and DE genes in resting MFM vs. control muscle consistent with increased connective tissue. There was also a >2-fold log_2_ increased expression of the basement membrane-associated gene *USH2A* in resting MFM vs. control muscle. Histologically, however, only a slight increase in endomysial connective tissue was apparent in two of the most severely affected MFM horses (most desmin aggregates). Regeneration was not apparent in MFM muscle based on HE and MHCd stains, and notably, *DLK1* was significantly downregulated in MFM vs. control horses. Ablation of *DLK1* in murine models results in impaired muscle regeneration (67).

**Fig. 6.**
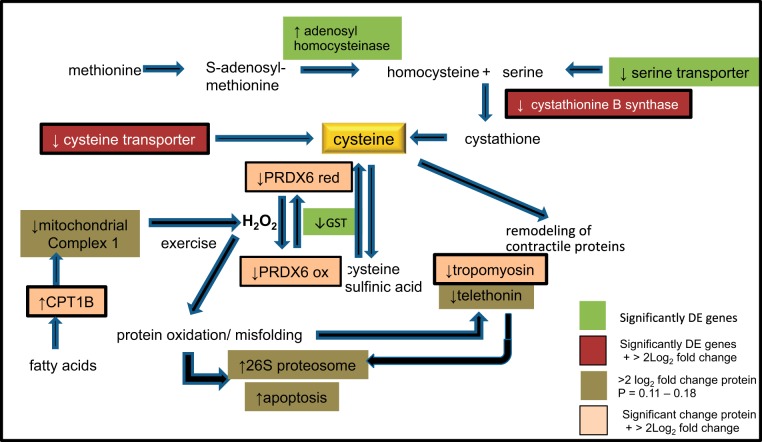
Schematic diagram of interrelationship among selected identified proteins and genes differentially expressed in MFM vs. control horse muscle at rest and after exercise. MFM horses have significantly higher activity of CPTB1 (3.5 log_2_ fold), which transports fat into mitochondria and lower expression of 2/24 subunits of mitochondrial Complex 1, which is a site of free radical generation. The 4.14 log_2_ fold lower content of cysteine-dependent peroxiredoxin 6 (PRDX6) in MFM vs. control muscle at rest and lower expression of enzymes required to synthesize cysteine following exercise indicated that the oxidoreductase pathway plays a central role in the pathogenesis of MFM. We hypothesize that protein misfolding, myofibrillar disarray, and aggregation of desmin in MFM horses arise from protein oxidation due to excessive generation of reactive oxygen species (H_2_O_2_), decreased postexercise cysteine synthesis, and depletion of cysteine-dependent antioxidants such as PRDX6.

A high proportion of high oxidative type 1 and 2A fibers in gluteal muscle is a feature of successful endurance racing Arabian horses and was found in the horses in the present study (49). This fiber type composition serves to delay fatigue by enhancing oxidation of free fatty acids during aerobic exercise and sparing muscle glycogen. Compared with control horses, MFM horses had a higher capacity to transport fatty acids into mitochondria for β-oxidation as indicated by the significantly higher content of carnitine palmitoyl transferase (↑3.5 log_2_ fold MFM), the enzyme responsible for transport of long chain fatty acids into mitochondria. β-oxidation of free fatty acids during aerobic exercise though energy efficient, is also a primary generator of reactive oxygen species (ROS). ROS are generated primarily via mitochondrial Complex I and III of the electron transport chain and must be reduced by specific enzymes to prevent cellular damage. In MFM horses, the content of two out of 24 identified Complex I subunit proteins was >2 log_2_ fold lower than in control horses (36). The potential to generate more ROS in MFM vs. control horses could arise from enhanced uptake of fatty acids into mitochondria in MFM horses, increased subsequent flux of electrons through Complex I and increased ROS produced because of an altered Complex I subunit composition (Fig. 6). Notably, oxidoreductase activity was one of the primary pathways dysregulated in MFM in the present study. To compound the effect of ROS generation further, MFM horses appeared to have a lower capacity to neutralize ROS than controls, with a significantly lower content of the antioxidant PRDX6 (↓4 log_2_ fold MFM) (Fig. 6).

PRDX6 is a widely distributed cytoplasmic cytoprotective nonseleno peroxidase that reduces hydrogen peroxides and quite uniquely, phospholipid hydroperoxides, by using a cysteine-containing active site (23). Glutathione S transferase, a cysteine selenoprotein that reduces PRDX6, was among the DE genes in MFM vs. control muscle (↓0.5 log_2_ fold change MFM) (23). While increased PRDX6 is described in a number of neurodegenerative diseases and some cancers, only one disease, alcoholic steatitis, reports decreased PRDX6 protein content with a much lesser decrement in PRDX6 than that found for MFM horses (1.6-fold vs. 18-fold) (47). A decrease in the amount of PRDX6 could arise from decreased synthesis or from irreversible cysteine oxidation to sulfinic (SO_2_H) and sulfonic acid (SO_3_H) with subsequent ubiquitination and degradation by the proteasome (42). Enhanced proteasomal degradation was suggested in proteomic data by 2.4 log_2_ fold upregulation of the 26S proteasome subunit in MFM vs. control muscle. Notably the only gene with >1.0-fold log_2_ upregulation comparing rest to after exercise in MFM horses was *GADD45G*, a gene that regulates DNA repair in response to stimuli such as oxidative stress (70).

Following exercise, a remarkably focused lower expression of genes involved in cysteine transport and biosynthesis was identified in MFM compared with control muscle (Fig. 6). Cysteine beta synthase (*CBS*) and a cysteine transporter had >1.8 log_2_ fold lower expression in postexercise MFM compared with postexercise control muscle. In addition, adenylhomocysteinase, a key enzyme involved in synthesizing cysteine from methionine, was one of only five genes/proteins common to the transcriptomic and proteomic data sets of MFM vs. control horses. Cysteine residues are among the most sensitive to oxidation relative to other amino acids, and alterations in the redox state of cysteine residues can modify protein structure and enzyme activity (14, 26). Cysteine oxidation can result in a diverse array of modifications, some of which are reversible in vivo through interactions with glutathione, PRDX, or thioredoxin (14). Reversible cysteine oxidative modifications include intramolecular disulfides, S-nitrosylation, S-glutathionylation, and sulfonic acid, whereas irreversible cysteine oxidative modifications include sulfinic and sulfonic acid (14). One of the functions of PRXD6 and glutathione is to prevent irreversible oxidation of cysteine (Fig. 6). A deficiency of intracellular cysteine and PRDX6 in skeletal muscle following exercise in MFM horses could impair synthesis of cysteine-dependent antioxidants such as PRDX6 and glutathione, which could further impair PRDX6 availability and lead to irreversible oxidation of cysteine, an essential protein in MFM muscle (Fig. 6). The possibility that MFM horses have an underlying defect in cysteine biosynthesis and the redox system is intriguing. Clearly, alterations in antioxidant function, cysteine uptake, and metabolism are worthy of further investigation in MFM horses.

Our initial focus in the present study was desmin and sarcomeric proteins because desmin aggregates and disruption of myofilaments and the Z-disc typify MFM in both equine and human cases (34, 53). Although desmin aggregates were evident in histologic sections of equine MFM muscle, significant differences in desmin protein or gene expression were not detected (63). One synonymous and several intronic variants were identified in the desmin gene; however, there was not a mutation that was consistently present in all MFM horses and absent in control horses. Thus, a mutation in desmin does not appear to be responsible for Arabian MFM. Myotilin, filamin C, LIM protein, and αβ-crystallin protein content were similar between MFM and control horses. Furthermore, only one protein, a 26S proteosomal subunit, overlapped with proteins identified in a proteomic analysis of human patients with mutations in desmin and filamin C (40). The lack of overlap with sarcomere proteins in equine vs. human MFM could be because the present study used whole muscle samples vs. laser capture isolation of myofibers with desmin aggregates in the human study (40). Alternatively, the findings in the present study could suggest that the basis for equine MFM differs from desmin or filamin C-related MFM.

Desmin is a major target for oxidation and nitrosylation, which is well described in human MFM (31). In fact, the antioxidants N-acetyl cysteine and α-tocopherol have been shown to prevent desmin aggregation in cultured cells that express a desmin mutation (8, 54). The role of oxidation in desmin-related myopathies is also highlighted by the fact that a myopathy once classified as MFM based on desmin aggregation, selenoprotein 1-related myopathy, was found to be caused by mutations in *SEPN1*, which is involved in oxidative homeostasis (3, 9, 21). Thus, there are several precedents for disturbances in oxidoreductase activity causing the type of desmin aggregation noted in horses with MFM.

Tropomyosin was the third significantly altered protein in MFM compared with control muscle. Lower tropomyosin (↓3.2 log_2_ fold MFM) content in MFM muscle could be the result of protein oxidation or alternatively, could reflect a primary underlying disorder (30, 35, 36). Decreased tropomyosin content is known to occur in limb girdle muscular dystrophy 2A and hereditary inclusion body myositis (24), (55), which have different histopathologic appearances compared with MFM in horses (63, 64). Mutations in the Z-disc protein MYOZ2 can cause myofibrillar disarray and hypertrophic cardiomyopathy and gene expression of MYOZ2 was significantly decreased in resting MFM vs. control horses (45). Muscle from horses with MFM also had significantly higher DE of a desmin cross-linking gene, desmoplakin, primarily expressed in cardiac muscle. Mutations in genes encoding other members of the plakin family, plectin and dystonin, disrupt the sarcomere in a fashion similar to that seen with MFM (37). Two elements involving the cytoskeleton (PDLIM1) and protein misfolding (HSPB7) were also common to the proteomic data set and the MFM vs. control DE transcripts after exercise. Deficiency of HSBP7 can cause myofibrillar disarray (32). Thus, tropomyosin, myozenin 2, desmoplakin, and HSPB7 are also candidates for further investigation in equine MFM.

The power of combining RNA-Seq and iTRAQ proteomic analysis in the presented study was highlighted by our ability to detect distinct but overlapping pathways such as DE of cysteine transporters and cysteine synthetic enzymes in RNA-Seq and cysteine-dependent PRDX6 content in proteomics. qRT-PCR was used to validate DE of those genes with >2-fold log_2_ expression and revealed similar direction of changes to RNA-Seq data. With iTRAQ, we identified >600 confidently expressed proteins in equine muscle, which is more than the 400 proteins identified in human myopathies (55) and more than the 540 proteins found in studies of porcine muscle (29). Of the 673 proteins identified, 543 had expression in both the transcriptome and proteome. Little association was found, however, between DE of the same gene ID in the transcriptome and general expression in the proteome, similar to previous studies of myopathies such as murine muscular dystrophy (50). This may reflect the fact that long-term changes in gene expression are required to alter protein expression and that altered protein expression can arise not only from increased synthesis but also from enhanced or abnormal degradation (28).

Identification of many proteins and genes in the present study was limited by the lack of annotation provided in the current EquCab2.86 reference genome. Identification of small proteins was also potentially limited in the present study by use of whole muscle homogenates containing substantial amounts of large contractile proteins that could impair detection of less abundant smaller proteins. We used homogenates so that we were able to examine sarcomere and cytoskeletal proteins, which were of interest due to their importance in MFM. Previous iTRAQ analyses have utilized *t*-tests without correction for multiple testing to compare protein concentrations (48, 71). We utilized stringent correction for multiple testing, resulting in identification of only three highly significant proteins. We also included those proteins with >2 log_2_ fold changes to fully evaluate pathways associated with MFM.

### Conclusions

In conclusion, this study defines for the first time a potential pathophysiological basis for an exertional myopathy in Arabian horses. Transcriptomic and proteomic profiles at rest and with exercise indicate that equine MFM is characterized by differential regulation of pathways involved in fatty acid metabolism, oxidoreductase activity, cysteine metabolism, sarcomere and cytoskeleton structure, as well as tissue regeneration and cell differentiation. A deficiency of cysteine-containing antioxidants and enhanced generation of ROS in MFM horses could lead to oxidative stress during exercise, oxidation of key proteins such as desmin and altered cysteine synthesis following endurance exercise.

## GRANTS

Support was provided by the Morris Animal Foundation Grant D14EQ-021. Support for C. J. Finno was provided by National Institutes of Health Grants 1K01OD-015134 and L40 TR-001136. The funding sources did not contribute to study design; in the collection, analysis and interpretation of data; in the writing of the report; or in the decision to submit the article for publication.

## DISCLOSURES

No conflicts of interest, financial or otherwise, are declared by the authors.

## AUTHOR CONTRIBUTIONS

S.J.V., E.C.M., and C.J.F. performed experiments; S.J.V., S.P., and C.J.F. analyzed data; S.J.V., S.P., and C.J.F. interpreted results of experiments; S.J.V. and S.P. prepared figures; S.J.V. drafted manuscript; S.J.V., S.P., E.C.M., and C.J.F. edited and revised manuscript; S.J.V., S.P., E.C.M., and C.J.F. approved final version of manuscript.
